# A Spike-Based mRNA Vaccine Encapsulated in Phospholipid 1,2-Dioleoyl-sn-Glycero-3-PhosphoEthanolamine Containing Lipid Nanoparticles Induced Potent B- and T-Cell Responses Associated with Protection Against SARS-CoV-2 Infection and COVID-19-like Symptoms in Hamsters

**DOI:** 10.3390/vaccines13010047

**Published:** 2025-01-08

**Authors:** Afshana Quadiri, Swayam Prakash, Latifa Zayou, Nisha Rajeswari Dhanushkodi, Amruth Chilukuri, Gemma Ryan, Kelly Wang, Hawa Vahed, Aziz A. Chentoufi, Lbachir BenMohamed

**Affiliations:** 1 Laboratory of Cellular and Molecular Immunology, Gavin Herbert Eye Institute, School of Medicine, University of California Irvine, Irvine, CA 92697, USA; aquadiri@hs.uci.edu (A.Q.); fswayamp@hs.uci.edu (S.P.); lzayou@hs.uci.edu (L.Z.); nishazone2000@gmail.com (N.R.D.); achilukuri@ucsd.edu (A.C.); hvahed@hs.uci.edu (H.V.); aalamich@hs.uci.edu (A.A.C.); 2 Precision Nanosystems Inc., Vancouver, BC V6P 6T7, Canada; gemma.ryan@cytiva.com (G.R.); kelly_wang@pall.com (K.W.); 3 Department of Vaccines and Immunotherapies, TechImmune LLC, University Lab Partners, Irvine, CA 92660, USA; 4 Division of Infectious Diseases and Hospitalist Program, Department of Medicine, School of Medicine, University of California Irvine, Irvine, CA 92697, USA; 5 Institute for Immunology, School of Medicine, University of California Irvine, Irvine, CA 92697, USA

**Keywords:** lipid nanoparticles, mRNA/LNP, COVID-19, phospholipid, DOPE (1,2-dioleoyl-sn-glycero-3-Phosphoethanolamine)

## Abstract

Background: Nucleoside-modified mRNA encapsulated in lipid nanoparticles (LNPs) have emerged as a promising vaccine strategy, especially for COVID-19. While the LNPs protect mRNA from degradation and efficiently deliver the mRNA to antigen-presenting cells, the effect of lipid composition on the immunogenicity and protective efficacy of mRNA/LNP vaccines is not well characterized. Studies on using the mRNA/LNP platform for vaccines have largely focused on the nucleic acid cargo with less attention paid to the LNP vehicle. Whether the composition and biophysical properties of LNPs impact vaccine performance remains to be fully elucidated. Methods: In the present study, we used SARS-CoV-2 Spike-mRNA as a prototype vaccine to study the effect of four different LNPs with various lipid compositions. Results: We demonstrate that when the same Spike-mRNA was delivered in the LNP4 formulation based on phospholipid 1,2-dioleoyl-sn-glycero-3-Phosphoethanolamine, it outperformed other LNPs (LNP1, LNP2, and LNP3) that are based on different lipids. Compared to the other three LNPs, LNP4 (i) enhanced the phenotypic and functional maturation of dendritic cells; (ii) induced strong T-cell responses; (iii) increased the secretion of proinflammatory cytokines and pro-follicular T helper (Tfh) cell cytokines; (iv) induced higher neutralization IgG titers; and (v) provided better protection against SARS-CoV-2 infection and COVID-19-like symptoms in the hamster model. Furthermore, we compared LNP-4 with the commercially available LNPs and found it to provide better T-cell immunity against COVID-19 in hamsters. Conclusion: This study suggests mRNA vaccines encapsulated in Phospholipid 1,2-Dioleoyl-sn-Glycero-3-PhosphoEthanolamine containing LNPs induced Potent B- and T cell immunity. The mechanisms by which Phospholipid 1,2-Dioleoyl-sn-Glycero-3-PhosphoEthanolamine-based LNPs may activate protective B and T cells are discussed.

## 1. Introduction

The development of safe and efficacious vaccination against diseases that cause substantial morbidity and mortality is the greatest contribution of any health intervention [1,2,3,4]. The beginning of the 21st century marked a revolution in molecular biology and provided insights into microbiology and immunology allowing a greater understanding of vaccine delivery systems [5,6,7]. Lipid nanoparticles (LNPs) have recently emerged as one of the most advanced vehicle platforms for the efficient in vivo delivery of nucleoside-modified mRNA vaccines, particularly for COVID-19 [8].

The use of LNPs in COVID-19 mRNA vaccines gained significant attention for delivering the mRNA to cells demonstrating several advantages over viral vectors, including low cytotoxicity and good immunogenicity, simple production, and great scalability [9,10]. mRNAs are inherently unstable and prone to rapid degradation by nucleases and self-hydrolysis. LNPs are efficient for mRNAs’ encapsulation, protect them from extracellular ribonucleases, and assist with in vivo and intracellular mRNA delivery [11,12]. Studies on using mRNA/LNP vaccines have largely focused on characterizing the nucleic acid cargos, with less attention being paid to LNP vehicles. While LNPs protect mRNA from degradation and efficiently deliver mRNAs in vivo to antigen-presenting cells, the effect of lipid composition and the biophysical properties of LNPs on the immunogenic and protective efficacy of mRNA/LNPA vaccines remain to be fully elucidated.

LNPs are spherical delivery vehicles that comprise four different lipid components: (i) ionizable lipids, which are primarily required for mRNA complexing; (ii) helper lipids, which enhance the properties of LNPs (stability and delivery efficiency); (iii) cholesterol, which provides structural stability to the LNPs (vehicle); and (iv) surface PEGylation with poly(ethylene glycol) (PEG) or PEG derivatives, which reduces host-immune recognition and improves systemic circulation [13]. These lipids provide several benefits, including formulation simplicity, self-assembly, biocompatibility, high bioavailability, and the potential to transport large payloads [13]. Ionizable lipids are the most essential components of LNPs, as they are neutral in the physiological pH environment but positively charged under low pH conditions. Ionizable lipids form complexes with the negatively charged mRNA and subsequently releases it in the cytosol [14]. The nucleic acid encapsulated in LNPs is delivered through absorption by the cell plasma membrane and the eventual uptake into the cell by endocytosis. After endocytosis, the nucleic acid is released inside the cell. The adsorption of LNPs is facilitated electrostatically because of the difference between the negatively charged cell membrane and the positively charged LNPs [15]. When the LNPs enter the cell, nucleic acid is released from the cationic carrier, and the LNPs’ charge is neutralized by anionic lipids found in the cells. This neutralization of the LNPs stops electrostatic attraction between the lipids and nucleic acids, disrupting the structure of the nanoparticles and causing a non-lamellar structure, thus delivering mRNAs with high mRNA encapsulation, greater stability, and pH-sensitive in vivo delivery [16].

Currently, there are several mRNA-based vaccines against SARS-CoV-2 that utilize different LNP compositions, of which two are Pfizer–BioNtech’s BNT162b2 and Moderna’s mRNA-1273 [17]. Pfizer–BioNtech uses an ionizable cationic lipid called ALC-0315, which they licensed from Acuitas. The nanoparticles used in Pfizer–BioNtech BNT162b2 are ALC-0315, ACL-0159, 1,2-stearoyl-sn-glycerol-3-phosphocholine (DSPC), and cholesterol. ALC-0159 is a polyethylene glycol conjugate, meaning it is a PEGylated lipid [18]. Moderna uses its own unique and proprietary ionizable cationic lipid called SM-102. The nanoparticles used in Moderna mRNA-1273 are 1,2-stearoyl-sn-glycerol-3-phosphocholine (DSPC), polyethylene glycol 200-dimyristoyl glycerol (PEG2000-DMG), cholesterol, and SM-102 [12,19]. In both mRNA-based vaccines, the lipids used are 1,2-stearoyl-sn-glycerol-3-phosphocholine (DSPC), cholesterol, a type of PEGylated lipid, and an ionizable cationic lipid. Modifications made to the cholesterol used in these mRNA-based vaccines increases the cellular uptake of LNPs [20]. The PEG-lipid, with its chemical structure of a hydrophilic and hydrophobic region, allows penetration across lipid membranes and influences the size of the particle [21]. The ionizable lipid design has undergone numerous improvements leading to much higher transfection potency. The first ionizable lipid reported AL1 synthesized for nucleic acid delivery, exhibited low therapeutic levels. Improvements to the lipid design resulted in nearly 8000-fold improvements in the therapeutic index [22]. Introducing a ketal group in the ionizable lipid led to 10% increase in gene silencing by LNPs [23].

The challenges remaining for mRNA/LNP vaccines involve the complexity associated with identifying the best formulation. The detailed mechanistic knowledge of how LNPs assist with the endocytosis of mRNA is still lacking. This makes improvements to the design and formulation of LNPs difficult. For most formulations, the bottleneck has not been identified, whether it is endocytosis, endosomal escape, stability of the mRNA, DC activation, or something different [24]. Much research has been performed to improve the endosomal escape and transfection efficiency of LNPs by modifying lipid compositions. However, compared to the extensive research conducted exploring different lipid formulations, the nanostructure of LNPs or the specific packing of lipids and nucleic acids into LNPs has not been fully investigated. Over the past 20 years, ionizable lipid design has undergone numerous improvements. The first ionizable lipid reported AL1, also known as DODAP, did not provide significant therapeutic levels of delivery unless high doses were administered. Improvements to the lipid design resulted in nearly 8000-fold improvements in the therapeutic index [25]. Interestingly, the ester-containing analog, was found to be ineffective while a ketal-containing compound exhibited 10-fold more activity than the effective dose required to achieve 50% gene silencing [20,25]. LNP size plays a critical role in delivery for larger nanoparticles, however research is required to understand whether and how LNP size influences mRNA delivery. A comparative evaluation of different LNPs is, therefore, required to determine the optimal parameters for the desired vaccination. In the present study, we investigated the pre-clinical efficacy of four different LNPs. The four LNPs (described in this study as LNP-1, LNP-2, LNP-3, and LNP-4) differed in their phospholipid compositions or ionizable lipid relative amount, which we found to significantly affect the degree of protection from COVID-19 disease.

In this study, we observed that LNP-4 that contains a phospholipid constituent named as 1,2-dioleoyl-sn-glycero-3-Phosphoethanolamine (DOPE) has higher potency at inducing better immune responses and better protection in hamsters upon SARS-CoV-2 infection. Compared to the other three LNPs, LNP-4 (i) enhanced the phenotypic and functional maturation of dendritic cells; (ii) induced strong T-cell responses; (iii) increased the secretion of proinflammatory cytokines and pro-follicular T helper (Tfh) cell cytokines; (iv) induced higher neutralization IgG titers; and (v) provided better protection against SARS-CoV-2 infection and COVID-19 in the hamster model. In addition, we compared LNP-4 with a well-established pre-optimized ionizable lipid mix for mRNA-LNP delivery known as GenVoy-ILM^TM^. The formulation developed by PNI has displayed vaccine stability and efficacy and has been used for both in vitro and in vivo deliveries of mRNA and self-amplifying RNA (saRNA) for infectious disease vaccine research [26,27,28,29]. In this study, we refer to GenVoy-ILM^TM^ as LNP-O (LNP “Off the shelf”). We observed that Spike mRNA encapsulated in LNP-4 elicited higher antibody titers compared to LNP-O upon immunization. In addition, LNP-4-immunized hamsters exhibited significantly higher T-cell responses as well as a display of activation markers on CD4+ and CD8+ T cells in comparison to LNP-O. The findings point to the need for understanding and adjusting lipid formulations to achieve a higher efficacy of LNP-mRNA-based vaccines.

## 2. Materials and Methods

**LNP formulation:** Four customized GenVoy-ILM-based lipid nanoparticles containing a common ionizable cationic lipid and with differing compositions of phospholipids, cholesterol, and stabilizer were provided by Precision NanoSystems (PNI) (currently Cytiva) for this project. The 4 LNPs differed in their phospholipid compositions or ionizable lipid relative amounts. LNPs with these compositions are available for purchase from PNI upon request using the identifiers IL00V02, IL00V03, IL00V29, and IL00V41. For the convenience of this study, we used LNP1, LNP2, LNP3, and LNP4 to identify IL00V02, IL00V03, IL00V29, and IL00V41, respectively. Each of these 4 LNPs comprised a unique phospholipid that is the second listed lipid component of the LNP. Different phospholipids are associated with the 4 LNPs as follows: (i) LNP1 (IL00V02) composed of DSPC (1,2-distearoyl-sn-glycero-3-phosphocholine), (ii) LNP2 (IL00V03) composed of DOPC (1,2-dioleoyl-sn-glycero-3-phosphocholine), (iii) LNP3 (IL00V29) composed of DPPC (dipalmitoylphosphatidylcholine), and (iv) LNP4 (IL00V41) composed of DOPE (1,2-dioleoyl-sn-glycero-3-phosphoethanolamine). While the ionizable phospholipid that was used in all the formulations was the same, the amount used between formulations varied. LNP3 (IL00V29) and LNP4 (IL00V41) both have a “higher” amount of ionizable lipid, but the phospholipid composition is different. A single ionizable lipid was used across all formulations. The ionizable lipid and its structure are proprietary to Cytiva. The physical properties associated with the four LNPs are further described in Table 1. Along with the four customized LNP formulations, we also used for comparison the “Off-the Shelf” available LNP formulation named GenVoy-ILM^TM^, which is a commercially available proprietary lipid-mix composition comprising DSPC:cholesterol:ionizable-lipid:stabilizer at 10:37.5:50:2.5 mol% for encapsulating nucleic acids, manufactured by Precision NanoSystems Inc (Vancouver, BC, Canada). In this article, this “Off-the Shelf” LNP formulation is called LNP-O.

To prepare the lipid encapsulation, the mRNA stock was first diluted in the PNI’s formulation buffer. Subsequently, the 4 GenVoy-ILM-based lipid mixes were prepared in ethanol at a 12.5 mM concentration. The LNP formulations were prepared on NanoAssemblr Ignite using a specific formulation scheme, which included a flow rate ratio (FRR) for the mRNA solution to lipid solution of 3:1; total flow rate (TFR) of 12 mL/min; start waste of 1 mL; and end waste of 0.05 mL. After the formulation, the post-chip formulations were diluted to a total 4X volume in sterile Ca^2+^ and Mg^2+^ free PBS. Amicon^®^ Centrifugal Filters (Merck KGaA, Darmstadt, Germany) with an MWCO of 30 KDa (LNP1, LNP2, and LNP3) or an MWCO of 100 KDa (LNP4) were used to concentrate formulations and perform ethanol removal and buffer exchange to 1X PBS (pH 7–7.3, Mg^2+^/Ca^2+^—free). mRNA LNPs were filtered manually through a 0.22 μm syringe filter using aseptic techniques and were stored at 4 °C. Finally, the particle size and polydispersity index (PDI) were analyzed using dynamic light scattering (DLS). RNA encapsulation efficiency and concentrations were determined using a RiboGreen plate-based assay.

**mRNA synthesis:** Sequences of the Spike antigen were derived from the SARS-CoV-2 Omicron sub-variant BA.2 (NCBI GenBank accession number OM617939). Nucleoside-modified mRNAs expressing the SARS-CoV-2 full-length-of-prefusion-stabilized Spike protein with two proline mutations (mRNA-S-2P (size: 3804 bp; nucleotide range: 21,504 bp–25,308 bp)) were synthesized by in vitro transcription using T7 RNA polymerase (MegaScript, Thermo Fisher Scientific, Waltham, MA, USA) on linearized plasmid templates, as previously reported [30]. A modified mRNA transcript with a full substitution of Pseudo-U was synthesized by TriLink Biotechnologies using proprietary CleanCap^®^ technology. The synthesized polyadenylated (80A) mRNAs were subjected to DNase and phosphatase treatment, followed by silica membrane purification. Finally, the synthesized mRNA was packaged as a 1.00 ± 6% mg/mL solution in 1 mM of sodium citrate, pH 6.4. Purified mRNA was analyzed by agarose gel electrophoresis and kept frozen at −20 °C.

**Human Samples**: Blood samples were obtained from healthy donors. Consenting adults were screened using a questionnaire determining their demographic information, medication usage, and comorbidities. Participants were excluded from any acquired immunodeficiency or immunomodulating medications (such as steroids or chemotherapy), pregnancy, history of cancer, history of cirrhosis or renal failure, or antibiotic use within 2 weeks of recruitment. Blood samples were taken from individuals aged 18–75 years. The experiments were approved by the Institutional Biosafety Committee at the University of California, Irvine (Protocol number BUA-R112). Written informed consent was obtained from all patients before inclusion.

**PBMC isolation:** The blood of anonymous healthy donors was diluted with RPMI1640. Peripheral blood mononuclear cells (PBMCs) were isolated by density centrifugation using percoll. After centrifugation, the interphase containing PBMCs was collected and washed twice with RPMI1640. The cells were cultured in tissue culture dishes and incubated at 37 °C, 5% CO_2_.

**Generation of human monocyte-derived dendritic cells (MoDCs):** The DCs in PBMC were allowed to adhere to the culture dish. On the next day, the medium was replaced with DC medium containing 800 U/mL of GM-CSF and 50 U/mL of IL4. On day 3 and day 5, a total of 4 mL of fresh DC medium supplemented with 800 U/mL of GM-CSF and 50 U/mL of IL4 was added. On day 7, immature as well as mature DCs were harvested. As moDCs adhere loosely to the culture dish, the use of EDTA or cell scrapers is not needed. Thus, the supernatant was flushed a couple of times to obtain moDCs. Afterward, moDCs were treated with different LNPs for 24–36 h. The cells were harvested and stained for FACS analysis. The supernatant was collected and analyzed for different cytokines.

**Flow cytometry:** The following anti-human antibodies were used for the flow cytometry assays: CD11c (clone BLY6—BD BioSciences), HLA-DR (clone G46-6—BD BioSciences), CD40 (clone 5C3—Invitrogen), CD86 (clone 2331 (FUN-1)—BD BioSciences), and CD83 (clone HB15e—BD BioSciences). For the surface stain, mAbs against cell markers were added to a total of 1 × 10^6^ cells in 1X PBS containing 1% FBS and 0.1% sodium azide (FACS buffer) for 30 min at 4 °C. The cells were washed with FACS buffer and finally suspended in 200 uL of FACS buffer. For each sample, 50,000 total events were acquired on the BD LSRII. Ab capture beads (BD Biosciences) were used as individual compensation tubes for each fluorophore in the experiment. We used fluorescence minus controls for each fluorophore to define positive and negative populations when initially developing staining protocols. Data analysis was performed using FlowJo version 10.10. Statistical analyses were performed using GraphPad Prism version 7.

**Hamster mRNA/LNP immunization and SARS-CoV-2 virus challenge:** The mRNA/LNP vaccines were evaluated in the outbred golden Syrian hamster model for protection against the SARS-CoV-2 USA/WA1/2020 strain. The Institutional Animal Care and Use Committee approved animal model usage experiments at the University of California, Irvine (Protocol number AUP-22-086). The recommendations are present in the Guide for the Care and Use of Laboratory Animals of the National Institutes of Health performed animal experiments. The total sample size of 6–8-week-old male Syrian golden hamsters used in this was 45. Hamsters divided into nine experimental groups were vaccinated with 1 µg (*n* = 20) and 3 µg (*n* = 20) of LNP1, LNP2, LNP3, and LNP4 encapsulating Spike protein intramuscularly in 100 μL doses into the posterior thigh muscle, twice (on day 0 and day 21). The mock group of hamsters was not immunized with any of the mRNA-LNP formulations. Four weeks after the first immunization, blood samples were collected from hamsters under isoflurane anesthesia and spun at 2000× *g* for 10 min to obtain serum. The hamsters were challenged intranasally with 100 µL of 1 × 10^5^ PFU of the SARS-CoV-2 USA/WA1/2020 strain diluted in sterile Dulbecco’s modified Eagle’s medium (DMEM). Weights were recorded daily until 14 days p.i. Oropharyngeal swabs were collected on days 2, 6, 10, 1and 4 in 500 µL of RNA later for virus titrations. The animals in each group were monitored daily for signs of disease and weighed until 14 days p.i.

In a separate experiment, we compared the efficacy of the LNP4 formulation with the commercially available GenVoy-ILM^TM^ (LNP “Off the shelf”). In this context, we used three groups of hamsters comprising immunization with (i) Spike-mRNA/LNP-4 (*n* = 6), (ii) Spike-mRNA/LNP-O (*n* = 6), and (iii) Mock (*n* = 6).

**QuantiFERON assay:** The whole heparinized blood sample was collected from each hamster for performing the QuantiFERON assay to determine the secretion of IFN-γ (QuantiFERON SARS-CoV-2, Qiagen). After overnight incubation with SARS-CoV2-specific peptides, supernatant was collected from LNP-stimulated samples in vitro at 12 and 24 h time points for IFN-γ analysis. The cytokine analysis was performed on 96-well flat bottom microplates. ELISA plates were coated with specific capture antibodies. The supernatant obtained from LNP-stimulated moDCs was tested without any dilutions. For hamster ELISA experiments, the sera were collected from blood isolated from immunized hamsters. ELISA was performed on sterile 96-well flat-bottom microplates coated with the Spike antigen in coating buffer overnight at 4 °C. The reaction was terminated by adding 1 M of H_2_SO_4_. The absorbance was measured at 450 nm.

**Enzyme-linked immunosorbent assay (ELISA):** Serum antibodies for SARS-CoV-2 proteins were detected by ELISA. The 96-well plates (Dynex Technologies, Chantilly, VA, USA) were coated with 100 ng of S protein per well at 4 °C overnight, and then washed three times with PBS and blocked with 3% BSA (in 0.1% PBST) for 2 h at 37 °C [31,32]. After blocking, the plates were incubated with serial dilutions of the sera (100 μL/well, in a two-fold dilution) for 2 h at 37 °C. The bound serum antibodies were detected with HRP-conjugated goat anti-mouse IgG and chromogenic substrate TMB (ThermoFisher, Waltham, MA, USA). The cut-off for seropositivity was set as the mean value plus three standard deviations (3SDs) in HBc-S control sera.

**Neutralization Assay:** Serum-neutralizing activity was examined, as previously reported in [33,34,35]. The assays were performed with Vero E6 cells (ATCC, CRL-1586) using the SARS-CoV-2 wild-type or Delta strains. Briefly, serum samples were heat-inactivated and three-fold serially diluted (initial dilution: 1:10), followed by incubation with 100 pfu of the wild-type SARS-CoV-2 (USA-WA1/2020) strain for 1 h at 37 °C. The serum–virus mixtures were placed onto a Vero E6 cell monolayer in 96-well plates for incubation for 1 h at 37 °C. The plates were washed with DMEM, and the monolayer cells were overlaid with 200 μL of minimum essential medium (MEM) containing 1% (*w*/*v*) of methylcellulose, 2% FBS, and 1% penicillin–streptomycin. Cells were then incubated for 24 h at 37 °C. Vero E6 monolayers were washed with PBS and fixed with 250 μL of pre-chilled 4% formaldehyde for 30 min at room temperature, followed by aspiration removal of the formaldehyde solution and washed twice with PBS. The cells were permeabilized using 0.3% (wt/vol) hydrogen peroxide in water. The cells were blocked using 5% non-fat dried milk followed by the addition of 100 μL/well of diluted anti-SARS-CoV-2 antibody (1:1000) to all wells on the microplates for 1–2 h at RT. This was followed by the addition of diluted anti-rabbit IgG conjugate (1/2000) for 1 h at RT. The plate was washed and developed by the addition of TrueBlue substrate, and the foci were counted using an ImmunoSpot analyzer (CTL, Cleveland, OH, USA). Each serum sample was tested in duplicates.

**Histology of animal lungs:** Hamster lungs were preserved in 10% neutral buffered formalin for 48 h before transferring to 70% ethanol. The tissue sections were then embedded in paraffin blocks and sectioned at 8 μm thicknesses. Slides were deparaffinized and rehydrated before staining for Hematoxylin and Eosin for routine immunopathology, as previously described [35,36,37].

**Virus titration in oropharyngeal swabs:** Throat swabs were analyzed for SARS-CoV-2-specific RNA by qRT-PCR. As recommended by the CDC, we used *ORF1ab-specific* primers (Forward-5′-CCCTGTGGGTTTTACACTTAA-3′ and Reverse-5′-ACGATTGTGCATCAGCTGA-3′) and a probe (6FAM-CCGTCTGCGGTATGTGGAAAGGTTATGG-BHQ) to detect the viral RNA level in oropharyngeal swabs. Briefly, 100 ng of the total nucleic acid eluate was added to a 20 µL total-volume reaction mixture (1x TaqPath 1-Step RT-qPCR Master Mix (Thermo Fisher Scientific, Waltham, MA, USA), with 0.9 mM of each primer and 0.2 mM of each probe. qRT-PCR was carried out using the ABI StepOnePlus thermocycler (Life Technologies, Grand Island, NY, USA).

**Statistical analysis:** The experimental groups were compared using the analysis of variance (ANOVA) applied using the multiple comparison-based Tukey test and Student’s *t*-test with GraphPad Prism version 10.1.0 (La Jolla, CA, USA). As we previously described, differences between the groups were identified by ANOVA and multiple comparison procedures [38,39]. The differences between the experimental groups were then identified and expressed as the mean ± SD. Results were considered statistically significant at *p* < 0.05.

## 3. Results

**1. Lipid nanoparticles with different compositions vary in their effect on the maturation and activation of dendritic cells:** Much of the immunobiology of dendritic cells (DCs) involves their role as antigen-presenting cells and in the activation of T cells. CD40 is upregulated on activated DCs and CD40L is expressed on activated T cells. The engagement of CD40 on DCs with CD40L induces positive signaling that leads to the expression of CD83/86 and the production of IL-12 in DCs.

**Figure 1:**
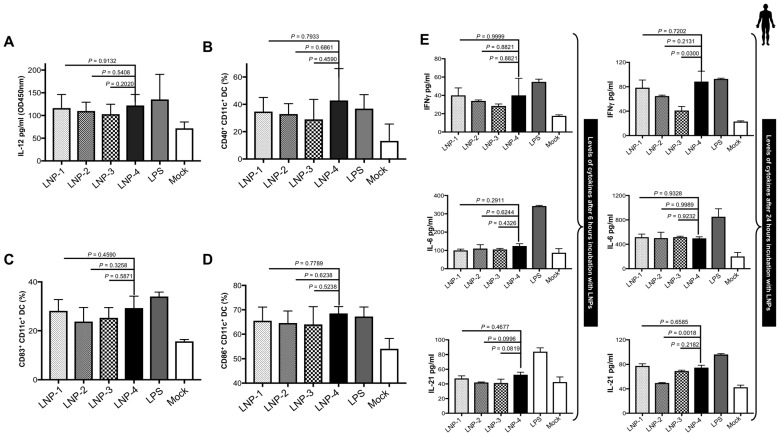
LNP stimulation and immunogenicity. (**A**) IL-12 secretion by monocyte-derived DCs after 24 h of stimulation with 1.2 μg/mL of different LNPs. (**B**) Activation status of monocyte-derived DCs as measured by CD40 expression after 24 h of stimulation with 1.2 μg/mL of different LNPs. (**C**,**D**) Expression of maturation markers CD83 (**C**) and CD86 (**D**) on DCs following stimulation with 1.2 μg/mL of different LNPs. (**E**) Dynamics of cytokines from PBMCS at 6 and 24 h after LNP stimulation.

We observed a similar phenomenon where the treatment of DCs with different LNPs led to the activation and maturation of DCs. Monocytes isolated from healthy participant’s PBMCs (*n* = 4; age range 24–75 yrs) were treated with GM-CSF/IL-4 for 6 days. On day 7, immature as well as mature DCs were harvested, followed by treatment with a dose of 1.2 μg/mL of four different LNPs for 24 h. We assessed the frequency of surface costimulatory and HLA marker-expressing cells in LNP-treated MDDCs compared to unstimulated cells after 24 h. We found that all LNPs significantly upregulated the percentage of IL12 and CD40-positive human MDDCs compared to the unstimulated control (Figure 1A,B). However, the upregulation was relatively higher in cultures treated with LNP-4 (Figure 1A,B). We also observed a significant upregulation in the maturation markers of MDDCs -CD83 and CD86 compared to the unstimulated controls (Figure 1C,D). However, the upregulation was relatively higher in cultures treated with LNP-4 (Figure 1C,D).

Next, we tested the production of cytokines following in vitro DC maturation after 6 h and 24 h of incubation with different LNPs. IL-6, IL-21, and IFN-γ were found to be markedly increased after 24 h in cultures stimulated with LNPs and this increase was relatively more pronounced in cultures stimulated with LNP-4 (Figure 1E). LNPs can induce pro-TFH cytokines, and key cytokines that are efficient in activating innate immune responses like IFN γ and LNP-4 displayed a greater ability compared to other LNPs.

**Figure 2:**
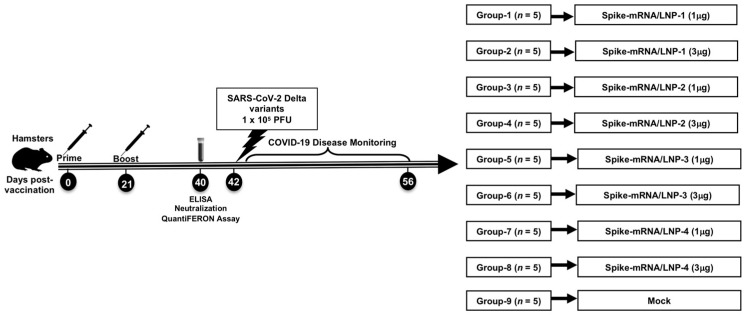
Schematic representation of experimental design. Syrian golden hamsters were vaccinated with 1 μg and 3 μg of the LNP1-4 encapsulating Spike protein intramuscularly twice (21 days apart). Four weeks after the first immunization, blood samples were collected from the hamsters under isoflurane anesthesia and spun to obtain serum. The hamsters were challenged with 1 × 10^5^ viral particles of the Delta strain diluted in sterile Dulbecco’s modified Eagle’s medium (DMEM). Weights were recorded daily until 14 DPI.

**2. Vaccination with mRNA-Spike encapsulated in LNP-4 demonstrated better protection against SARS-CoV-2:** We sought to assess the in vivo protection efficacy of mRNA-Spike LNPs in the well-established SARS-CoV-2 hamster model using the Delta strain. Groups of 8-week-old Syrian golden hamsters were i.m. administrated with either of the two doses of different (1 µg and 3 µg) concentrations of mRNA-Spike LNPs, followed by intranasal (i.n.) challenge with a sub-lethal dose (1 × 10^5^ pfu) of the SARS-CoV-2 Delta variant at 14 days following the second immunization (Figure 2).

**Figure 3:**
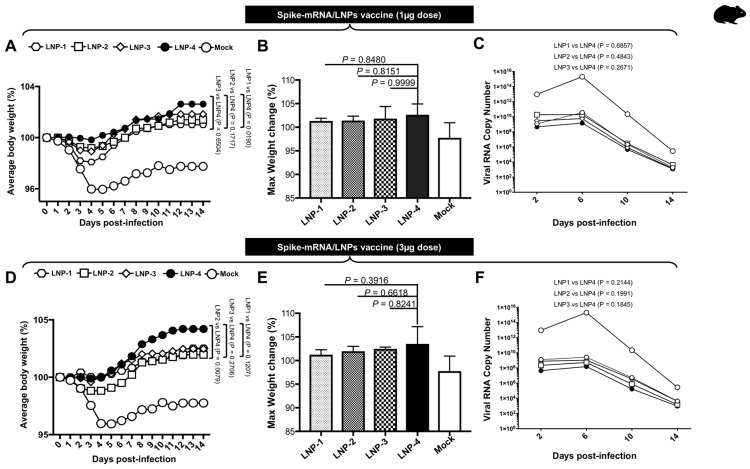
Vaccination of Syrian hamster using mRNA-Spike encapsulated in different LNPs followed by infection with the Delta variant: (**A**) mean body weight changes following immunization (1 μg dose) and infection of hamsters with the SARS-CoV-2 Delta variant, along with weight change in uninfected control hamsters. (**B**) Mean of weight gain in hamsters at 14 DPI following immunization (1 μg dose) and infection with the SARS-CoV-2 Delta variant. (**C**) SARS-CoV-2 viral RNA copy number comparison among hamsters immunized at 1 µg with mRNA-Spike-encapsulated LNPs. (**D**) Mean body weight changes following immunization (3 μg dose) and infection of hamsters with the SARS-CoV-2 Delta variant, along with weight change in uninfected control hamsters. (**E**) Mean of weight gain in hamsters at 14 DPI following immunization (3 μg dose) and infection with the SARS-CoV-2 Delta variant. (**F**) SARS-CoV-2 viral RNA copy number comparison among hamsters immunized at 1 µg with mRNA-Spike-encapsulated LNPs.

Weight loss analysis showed that all hamsters in the mock group lost body weight and displayed a mean reduction of 4–5% of body weight by day 4 (Figure 3A,D). Immunized hamsters showed a mean increase of 2–3% (1 µg) and 2–4% (3 µg) in body weight (Figure 3A,D). However, hamsters immunized with LNP-4 demonstrated better weight gain and overall maximum weight (Figure 3B,E). The viral titration data show relatively decreased viral RNA copy numbers for hamsters immunized with LNP4 at 1 µg (Figure 3C) and 3 µg (Figure 3F) in comparison to other LNPs.

**Figure 4:**
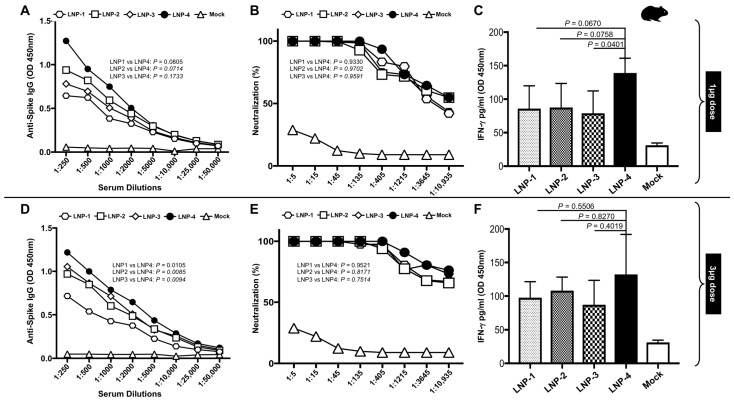
SARS-CoV-2-specific immune analysis by ELISA, neutralization, and QuantiFERON assays for select LNP formulations. (**A**) Spike-specific serum immunoglobulin (Ig) G or A binding antibody responses obtained from hamsters immunized with 1 μg of Spike-mRNA/LNP as measured by enzyme-linked immunosorbent assay (ELISA). (**B**) Serum-neutralizing antibody titers obtained from hamsters immunized with 1 μg of Spike-mRNA/LNP as measured by a plaque reduction neutralization test. (**C**) QuantiFERON assay to determine the induction of IFN-γ cytokine in peripheral blood of hamsters immunized with 1 μg of Spike-mRNA/LNP upon stimulation with Spike peptides. (**D**) Spike-specific serum immunoglobulin (Ig) G or A binding antibody responses obtained from hamsters immunized with 3 μg of Spike-mRNA/LNP as measured by the enzyme-linked immunosorbent assay (ELISA). (**E**) Serum-neutralizing antibody titers obtained from hamsters immunized with 1 μg of Spike-mRNA/LNP as measured by a plaque reduction neutralization test. (**F**) QuantiFERON assay to determine the induction of IFN-γ cytokine in the peripheral blood of hamsters immunized with 3 μg of Spike-mRNA/LNP upon stimulation with Spike peptides.

**3. Vaccination with mRNA-Spike encapsulated in LNP-4 induces high IgG titer and neutralizing antibodies:** To assess the immunogenic potential of mRNA/LNP vaccines formulated with four different LNP compositions (Table 1), Syrian golden hamsters (n = 5 per group) were vaccinated three weeks apart with two doses of either mRNA/LNP vaccines i.m. Immunogenicity was assessed at approximately 1 week after dose 2 (Day 28); Spike-specific serum immunoglobulin (Ig) G or A binding antibody responses were measured by enzyme-linked immunosorbent assay (ELISA) and serum neutralizing antibody titers were measured by a plaque reduction neutralization test (PRNT). Spike mRNA encapsulated in LNP-4 immunization elicited higher antibody titers followed by LNP 3, LNP-2, and LNP-1 (Figure 4A,D). LNP-1 displayed lower Spike-specific binding titers. In addition to S-specific binding titers, neutralizing antibody responses in sera were evaluated. LNP-4 displayed better neutralizing potential, followed by LNP-1 and LNP-2. LNP-3 displayed a lower neutralizing potential (Figure 4B,E).

**4. Vaccination with mRNA-Spike encapsulated in LNP-4 induced a higher level of Interferon-γ:** The heparinized, fresh, whole blood collected from each hamster immunized with different LNPs was incubated with SARS-CoV2 Spike-specific peptides for 24 h. The presence of IFN-γ in the plasma samples was determined by ELISA. Peripheral blood obtained from hamsters immunized with LNP-4 showed a higher production of IFN-γ in vitro followed by hamsters immunized with LNP-2, LNP-3, and LNP-1 (Figure 4C,F).

**Figure 5:**
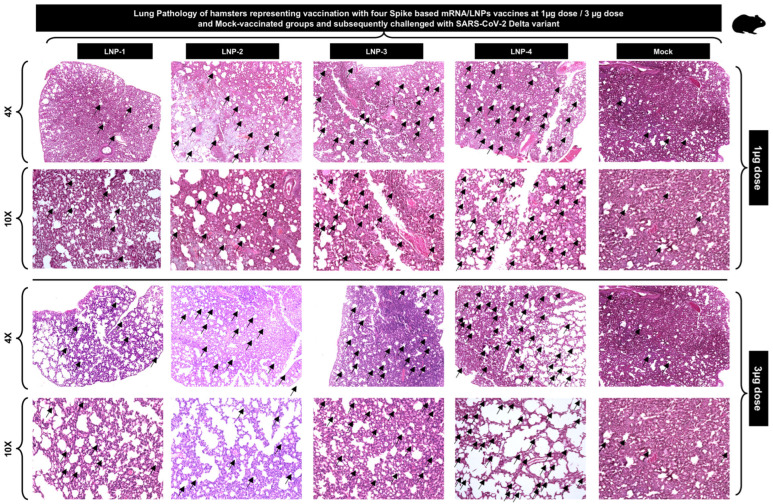
Histopathology and immunohistochemistry of the lungs from vaccinated and mock-vaccinated golden hamsters show reduced lung pathology in LNP-4 mRNA-LNP-based vaccinated hamsters. Representative images of Hematoxylin and Eosin (H&E) staining of the lungs harvested on day 14 p.i. from golden hamsters vaccinated with our novel COVID-19 vaccine formulated in different LNPs (LNP-1, LNP-2, LNP-3, and LNP-4) and mock-vaccinated hamsters. Hamsters were either vaccinated with individual mRNA-LNP COVID-19 vaccines at 1 μg or 3 μg doses. Reduced lung pathology is indicated with a higher number of lung vacuoles (marked with black arrows) and lower degree of hemorrhages. Images were captured at 4× and 10× resolutions.

**5. Vaccination with mRNA-Spike encapsulated in LNP-4 confers protection against SARS-CoV-2 Delta variants:** We next determined the protective efficacy mRNA/LNP vaccines that incorporate the Spike antigen against the highly pathogenic Delta variant (B.1.617.2). Hematoxylin and Eosin staining of lung sections at day 14 p.i. showed a significant reduction in COVID-19-related lung pathology in the hamsters vaccinated with mRNA/LNP vaccines and LNP-4 compared to mock-vaccinated mice (Figure 5). This reduction in lung pathology was observed at a higher level for hamsters vaccinated with 3 μg followed by hamsters vaccinated with a 1 μg dose of the LNP4 composition of the COVID-19 vaccine (Figure 5). Taken together, these results indicate that vaccination with our novel COVID-19 vaccine formulated with LNP-4 that constitutes phospholipid DOPE reduces COVID-19-related lung pathology following infection with the pathogenic SARS-CoV-2 Delta variant.

**Figure 6:**
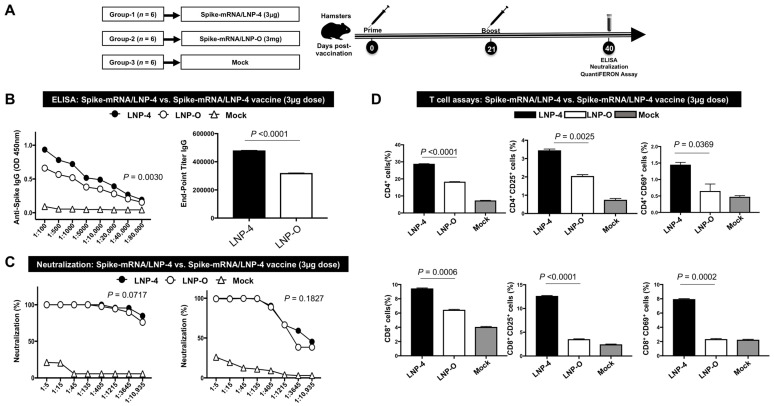
Immunogenicity comparison of LNP-4 with off-the-shelf LNP (LNP-O): (**A**) Schematic of experimental design. Syrian golden hamsters were vaccinated with 3 μg of LNP4 and LNP-O-encapsulating Spike protein intramuscularly twice (21 days apart). Four weeks after the first immunization, blood samples were collected from hamsters under isoflurane anesthesia and spun to obtain serum. (**B**) Spike-specific serum immunoglobulin (Ig) G or A binding antibody responses obtained from hamsters immunized with 3 μg of Spike-mRNA/LNP (LNP-4 vs. LNP-O) as measured by ELISA. (**C**) Serum-neutralizing antibody titers obtained from hamsters immunized with 3 μg of Spike-mRNA/LNP (LNP-4 vs. LNP-O) as measured by a plaque reduction neutralization test. (**D**) Analysis of T-cell responses in the peripheral blood of hamsters immunized with 3 μg of Spike-mRNA/LNP (LNP-4 vs. LNP-O).

**6. Vaccination with mRNA-Spike encapsulated in LNP-4 induced strong B- and T-cell responses compared to mRNA-Spike encapsulated in LNP-O:** To assess the immunogenic potential of LNP-4 in comparison to the well-established LNP formulation LNP-O (GeneVoy-ILM^TM^), mRNA-Spike was formulated in two different LNP compositions. Syrian golden hamsters (n = 6 per group) were vaccinated three weeks apart with two doses of either mRNA/LNP vaccines i.m. (Figure 6A). Immunogenicity was assessed at approximately 1 week after dose 2 (Day 28). Spike-specific serum immunoglobulin (Ig) G or A binding antibody responses were measured by enzyme-linked immunosorbent assay (ELISA) and serum-neutralizing antibody titers were measured by a plaque reduction neutralization test (PRNT). Spike mRNA encapsulated in LNP-4 immunization elicited higher antibody titers compared to LNP-O (Figure 6B). LNP-O displayed lower S-specific binding titers. In addition to S-specific binding titers, neutralizing antibody responses in sera were evaluated. LNP-4 displayed a slightly better neutralizing potential compared to LNP-O (Figure 6C). LNP-O displayed a slightly lower neutralizing potential. The heparinized, fresh, whole blood collected from each hamster immunized with different LNP-4 vs. LNP-O levels was analyzed for T-cell responses. LNP-4-immunized blood showed significantly better overall T-cell responses as well as a display of activation markers on CD4^+^ and CD8^+^ T cells (Figure 6D).

## 4. Discussion

The need for mRNA encapsulation arises from the fragility of mRNA as a standalone. mRNA molecules are inherently unstable and can be easily degraded by both extracellular nucleases and cellular enzymes. The immune system perceives mRNA as a foreign substance and tries to eliminate it when it is injected into the body. Even before the mRNA reaches the target cells, where it can be translated into proteins, this occurs very quickly. To limit the degradation and chemical reactions that result in the degradation of modified mRNA, current mRNA-based vaccines targeted against SARS-CoV-2 must be stored at extremely cold temperatures. In contrast, encapsulating mRNA in lipid nanoparticles (LNPs) protects it from degradation and immune recognition, allowing it to reach the target cells and be translated into proteins. LNPs in mRNA-based vaccines are designed to fuse with the cell membrane, allowing the encapsulated mRNA to enter the cell. A viral protein is subsequently synthesized by the ribosomes from the mRNA, which is subsequently presented on the cell surface for immune recognition. The vaccine can achieve a stronger immune response when the mRNA is encapsulated in LNPs rather than injected alone. In addition to prolonging the duration of protein expression, LNPs also provide the immune system more time to recognize and respond to viral proteins [40].

LNPs typically consist of a lipid core matrix that solubilizes the mRNA, as well as stabilizing surfactants or emulsifiers. The lipid core matrix of LNPs is usually composed of a mixture of lipids, including phospholipids, cholesterol, and other lipids, such as triglycerides, diglycerides, monoglycerides, and fatty acids [41]. These lipids include an ionizable cationic lipid whose positive charge can bind to the negatively charged mRNA, a PEGylated lipid meant for stability, and a phospholipid along with cholesterol designed for structural support. The lipid components facilitate the LNPs containing mRNA to enter a cell through an endosome. When the LNP is inside the acidic endosome, the ionizable lipids become positively charged and help to release the LNP and modified mRNA into the cell’s cytoplasm. The modified mRNA, now free, is translated by the ribosomes to create proteins. mRNA-encoded antigens more closely resemble the structure and presentation of viral proteins expressed during a natural infection. mRNA vaccines are vector-less and thus can avoid the potential for diminished immunogenicity with repeat dosing sometimes observed with vector-based vaccines. Moreover, mRNA vaccines for respiratory pathogens, such as SARS-CoV-2, have been established, demonstrating robust immune responses and high real-world effectiveness against disease. However, the most effective LNPs for vaccination need to be uncovered and investigated further.

In the present study, we underline the crucial role of delivery systems, i.e., lipid nanoparticles, in the efficacy of vaccines. LNPs improve the pharmacokinetics of the mRNA vaccine, such as its distribution and stability in the body, which can enhance the vaccine’s overall efficacy. However, LNPs need to be tailored with a specific lipid composition, which can maximize the effective immune response by promoting certain pathways of the immune system. Therefore, in this preclinical study, using SARS-CoV-2 as a model pathogen, the immunogenicity and protective efficacy of different LNPs encoding the SARS-CoV-2 specific Spike were evaluated. In this study, we expand upon the effect of LNP formulation on the induction of immune responses. Studies have shown that there is an intrinsic ability of LNPs to promote IL-6 secretion in mice and subsequent T_FH_ cell induction [42]. Here, we show that one specific LNP formulation (LNP4) induced higher maturation and the activation of DCs as measured by the frequency of co-stimulatory surface receptors and the production of cytokines and chemokines. By enhancing APC maturation and cytokine production, LNP-4 likely promotes a stronger and more durable immune response to the vaccine antigen compared to other LNPs. Additionally, a two-dose primary i.m. vaccination regimen of mRNA (Spike)-LNP elicited systemic immune responses and resulted in lower SARS-CoV-2 infection levels and disease severity versus mock-vaccinated controls after the viral challenge. Vaccination with mRNA/LNP4 showed improved protection against SARS-CoV-2. Based on the findings from this study, we used LNP-4 while formulating all our mRNA/LNP-based COVID-19 vaccines, which we found to be highly efficient in providing antigenicity and immunity against multiple SARS-CoV-2 variants of concern [43].

We observed that using LNP-4 that contains a phospholipid constituent named 1,2-dioleoyl-sn-glycero-3-Phosphoethanolamine (DOPE) has higher potency at inducing better immune responses and better protection in hamsters upon SARS-CoV-2 infection. In addition, we also assessed the immunogenic potential of LNP-4 in comparison to a commercially available (“off the shelf”) LNP, which we have referred to as LNP-O. We observed that Spike mRNA encapsulated in LNP-4 elicited higher antibody titers compared to LNP-O. LNP-O displayed lower S-specific binding titers. The heparinized, fresh, whole blood collected from each hamster immunized with different LNP-4 vs. LNP-O samples was analyzed for T-cell responses. LNP-4 immunized blood showed significantly better overall T-cell responses as well as a display of activation markers on CD4^+^ and CD8^+^ T cells.

The biophysical parameters of LNP largely impact its immunogenicity, and an understanding of the same is an important step for enabling the rapid development of potent mRNA/LNP vaccines. Different mRNA vaccine formulations developed for protection against infectious conditions have entered clinical studies to evaluate their effectiveness. The size of LNPs for example can have an impact on their immunogenicity, as demonstrated by studies such as the one conducted by Hassett et al. and Brewer et al. [44]. In the study by Hassett et al., the researchers investigated the effect of different biophysical factors on the immunogenicity of a cytomegalovirus (CMV) mRNA/LNP vaccine in mice. They found that LNP size had the strongest correlation with immunogenicity, with larger LNPs (up to 100 nm) leading to higher antibody titers. Similarly, Brewer et al. evaluated the antibody response to ovalbumin (OVA)-loaded liposomes of different sizes (100, 155, 225, and 560 nm) in mice [45] and found that smaller vesicles elicited higher levels of IgG1, which is indicative of a Th2-type immune response. In the present study, among the studied four LNPs, we have shown that LNP4 (IL00V41), which comprised DOPE phospholipid and having a relatively smaller size than other LNPs, provides better protection than other LNPs composed of DSPC, DOPC, and DPPC (Table 1). It is imperative to mention that this slight difference in size affects the immune response [44]. The finding from this study highlights the importance of focusing on incremental improvements, such as tweaking a single component like the phospholipid type or particle size, rather than attempting to overhaul multiple variables simultaneously. These small, deliberate refinements can collectively lead to significant advancements over time. Most importantly, these experiments formed our basis of LNP selection, and we used LNP-4 for antigen screening against SARS-CoV-2 [43].

Furthermore, we observed that vaccination with mRNA encoding the Spike protein of SARS-CoV-2 encapsulated in LNP-4 induces high levels of IgG antibodies and neutralizing antibodies against the virus. Additionally, peripheral blood obtained from hamsters immunized with LNP-4 showed a higher production of IFN-γ followed by hamsters immunized with other LNPs. LNP4 (IL00V41) comprising DOPE (1,2-dioleoyl-sn-glycero-3-phosphoethanolamine) and the amount of ionizable lipid used in the formulations were “higher” for LNP4 (IL00V41). Because the same antigen (Spike) and same dosing/immunization regimen were used in this study, we established the protective efficacy of various vaccine formulations. However, the mechanism of action of LNPs was not examined in this study. Nevertheless, our study points out the importance of the development and screening/testing of LNP formulations for favorable immunostimulatory profiles in studies such as these. The size and phospholipid dependence of LNPs for their efficacy are areas of ongoing research, with many aspects not fully understood. The role of specific phospholipid mixtures and their off-target effects are not fully understood. Future studies will address these important aspects.

## 5. Conclusions

Identifying the optimal formulation of lipid nanoparticles is challenging, as it depends on the specific molecule being encapsulated. The size and lipid composition need to be tailored to enhance delivery efficiency and stability. In the present case of mRNA vaccines for SARS-CoV-2, four types of LNPs were used. We slightly modified their formulations to determine the most effective LNP to be used in our mRNA-LNP vaccine. Based on the outcomes of this study, we used LNP4 to formulate our broad-spectrum multi-antigen mRNA/LNP-based pan-coronavirus vaccine. Our findings reveal that LNP4, with a relatively small particle size, contained DOPE and the highest proportion of ionizable lipids, significantly improving immune efficacy.

## Figures and Tables

**Table 1:** Physical properties associated with the LNPs.

LNP	Precision Nanosystems Nomenclature	Particle Size (d.nm)	PDI	Encapsulation Efficiency (%)	Zeta Potential (mV)
LNP-1	IL00V02	96	0.165	97.7	−10
LNP-2	IL00V03	101	0.158	97.3	−0.5
LNP-3	IL00V29	87	0.160	97.6	−7
LNP-4	IL00V41	94	0.154	98.4	−3

LNP: Lipid nanoparticle; PDI: polydispersity index; IL: ionizable lipid.

## Data Availability

Data are contained within the article.
